# Characterization of the complete chloroplast genome sequence of *Galinsoga parviflora* and its phylogenetic implications

**DOI:** 10.1080/23802359.2019.1623106

**Published:** 2019-07-10

**Authors:** Zhiyong Zhang, Ying Chen, Xuebo Jiang, Piao Zhu, Yanling Zeng, Tianming Tang, Ling Li

**Affiliations:** College of Life Sciences, Neijiang Normal University, Neijiang, China

**Keywords:** *Galinsoga parviflora*, chloroplast genome, phylogenetic analysis, weed

## Abstract

*Galinsoga parviflora* is an invasive weed in southwest of Chinese agricultural systems and commonly used as medicine and food. In this study, the complete chloroplast genome of the *G. parviflora* was assembled from the whole genome Illumina sequencing data. The circular genome is 151,811 bp in size, which composed of one large single-copy (LSC) and one small single-copy (SSC) regions of 83,594 bp and 18,141 bp, respectively, and separated by a pair of inverted repeat (IR) regions of 25,038 bp each. It encodes a total of 113 gene species (80 protein-coding, 29 tRNA, and four rRNA species), in which 19 of them with double copies. The overall GC content is 37.7% while the GC content of the LSC, SSC, and IR regions are 35.8%, 31.3%, and 43.1%, separately. Phylogenetic analysis indicated that *Galinsoga parviflora* was closely related to *Galinsoga quadriradiata*.

*Galinsoga parviflora* is one of the important species of the genus *Galinsoga* within the family Asteraceae (Asterales) (Ferheen et al. 2009; Ali et al. 2017). It is commonly found in Southwest of China such as Yunnan, Guizhou, and Sichuan Provinces (Pan et al. 2007). Although it is considered an invasive weed, the plant usually can be utilized as medicinal herb for wound healing as well as for the treatment of blood coagulation problems, cold, flu, toothache, and dermatological and eye diseases due to the presence of diverse secondary metabolites (Pan et al. 2007; Ali et al. 2017). The plant is full of essential oil containing bioactive compounds (Pino et al. 2010). So far, 38 compounds classified into seven categories (flavonoids, aromatic esters, diterpenoids, caffeic acid derivatives, steroids, phenolic acid derivatives, and miscellaneous compounds) have been isolated from *G. parviflora* (Ali et al. 2017). It has been reported that the plant had antibacterial (Matu and Van Staden 2003; Damalas, 2008; Pino et al. 2010), antifungal (Ali et al. 2017), anti-inflammatory (Matu and Van Staden 2003; Damalas, 2008), antioxidant (Chipurura et al. 2009; Bazylko et al. 2012; Bazylko et al. 2015), hepatoprotective (Mostafa et al. 2013), and hypoglycemic activity (Mostafa et al. 2013; Ali et al. 2017).

To facilitate its genetic research and contribute to its utilization, in this study, the complete chloroplast genome of the *G. parviflora* was assembled from the whole genome Illumina sequencing data. Phylogenetic analysis was conducted, which will be useful for further studies on its chloroplast genetic engineering.

Total genomic DNA was isolated from fresh leaves of an individual of *G. parviflora* from Sichuan province in southwest of China, located at 101°53′44″E, 30°52′44″N, and was stored in the Herbarium of Neijiang Normal University (accession number: 20190211GP03). The Illumina sequencing was conducted on Illumina HiSeq X Ten platform in Beijing Novogene Bioinformatics Technology Co., Ltd (Beijing, China). The complete chloroplast genome was assembled using the baiting and iterative mapping approach（Hahn et al. 2013), with that of its congener *Galinsoga quadriradiata* (GenBank accession number KX752097) (Wang et al. 2018) as the initial reference genome. The annotated genomic sequence has been submitted to GenBank with the accession number MK737938.

The circular chloroplast genome of *G. parviflora* was 151,811bp in size, which comprised of one LSC and one SSC regions of 83,594 bp and 18,141 bp, respectively, and separated by a pair of IR regions of 25,038 bp each. It encodes a total of 113 genes (80 protein-coding, 29 tRNA, and four rRNA species), in which 19 of them with double copies. Intron-exon structure analysis indicated that 16 genes (10 protein-coding genes and six tRNA genes) contained intron, in which of them two protein-coding genes (clpP and ycf3) had two introns while the others had one intron. The total GC content is 37.7%, while the corresponding values of the LSC, SSC, and IR regions are 35.8%, 31.3%, and 43.1%, separately.

To identify the phylogenetic position of *G. parviflora*, phylogenetic analysis was conducted. The maximum-likelihood (ML) phylogenetic tree was generated using species within the family Asteraceae by MEGA 7.0 (Kumar et al. 2016), which showed the position of *G. parviflora* was situated as the sister of *Galinsoga quadriradiata* in Asteraceae (Figure 1). The results indicated that *G. parviflora* and the other 10 species were clustered into a clade. Our findings will provide a foundation for further investigation of chloroplast genome evolution in *Galinsoga*.

**Figure 1.**
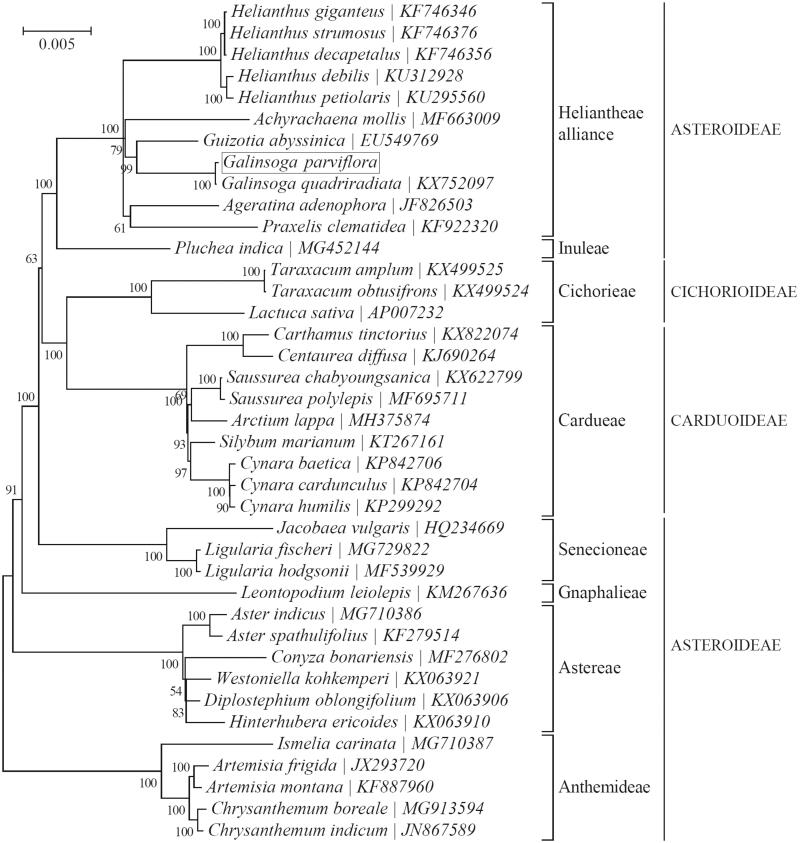
Phylogeny of 39 species within the family Asteraceae based on the maximum-likelihood (ML) of the concatenated chloroplast protein-coding sequences. The GTR + G + I model was employed as the best-fit nucleotide substitution model as suggested by MEGA7. The position of *Galinsoga parviflora* (GenBank accession number: MK737938) is shown in a box.

## References

[CIT0001] Ali S, Zameer S, Yaqoob M 2017 Ethnobotanical, phytochemical and pharmacological properties of *Galinsoga parviflora* (Asteraceae): a review. Trop J Pharm Res. 12:3023–3033.

[CIT0002] Bazylko A, Stolarczyk M, Derwińska M, et al. 2012 Determination of antioxidant activity of extracts and fractions obtained from *Galinsoga parviflora* and *Galinsoga quadriradiata*, and a qualitative study of the most active fractions using TLC and HPLC methods. Nat Prod Res. 17:1584–1593. 10.1080/14786419.2011.582469 22085305

[CIT0003] Bazylko A, Borzym J, Parzonko A 2015 Determination of in vitro antioxidant and UV-protecting activity of aqueous and ethanolic extracts from *Galinsoga parviflora* and *Galinsoga quadriradiata* herb. J Photochem Photobiol B Biol. 149:189–195. 10.1016/j.jphotobiol.2015.06.010 26092182

[CIT0004] Chipurura B, Muchuweti M, Parawira W, et al. 2009 An assessment of the phenolic content, composition and antioxidant capacity of *Bidens pilosa*, *Cleome gynandra*, *Corchorus olitorius*, *Galinsoga parviflora* and *Amaranthus hybridus*. I All Africa Horticultural Congress 911; p. 417–426.

[CIT0005] Damalas CA 2008 Distribution, biology, and agricultural importance of *Galinsoga parviflora* (Asteraceae). Weed Biol Manag. 3:147–153.

[CIT0006] Ferheen S, Aziz-Ur-Rehman AN, et al. 2009 Galinsosides A and B, bioactive flavanone glucosides from *Galinsoga parviflora*. J Enzyme Inhib Med Chem. 5:1128–1132. 10.1080/14756360802667688 19772485

[CIT0007] Hahn C, Bachmann L, Chevreux B 2013 Reconstructing mitochondrial genomes directly from genomic next-generation sequencing reads-a baiting and iterative mapping approach. Nucleic Acids Res. 41:e129. 23661685 10.1093/nar/gkt371 PMC3711436

[CIT0008] Kumar S, Stecher G, Tamura K 2016 MEGA7: molecular evolutionary genetics analysis Version 7.0 for bigger datasets. Mol Biol Evol. 33:1870–1874. 27004904 10.1093/molbev/msw054 PMC8210823

[CIT0009] Matu EN, Van Staden J 2003 Antibacterial and anti-inflammatory activities of some plants used for medicinal purposes in Kenya. J Ethnopharmacol. 1:35–41. 10.1016/s0378-8741(03)00107-7 12787952

[CIT0010] Mostafa I, El-aziz EA, Hafez S, et al. 2013 Chemical constituents and biological activities of *Galinsoga parviflora* Cav. (Astraceae) from Egypt. Z Naturforsch C Bio Sci. 7–8:285–292. 24066513

[CIT0011] Pan Z, Zhao L, Huang R, et al. 2007 Terpenes and sterols from *Galinsoga parviflora*. J Yunnan Univ (Nat Sci). 6:613–616.

[CIT0012] Pino JA, Gaviria M, Quevedo-Vega J, et al. 2010 Essential oil of *Galinsoga parviflora* leaves from Colombia. Nat Prod Commun. 11:1831–1832. 21213993

[CIT0013] Wang XY, Zhou ZS, Liu G, et al. 2018 Characterization of the complete chloroplast genome of the invasive weed *Galinsoga quadriradiata* (Asterales: Asteraceae). Conserv Genet Res. 1:89–92.

